# Controlling Fractional Free Volume, Transport, and Co-Transport of Alcohols and Carboxylate Salts in PEGDA Membranes

**DOI:** 10.3390/membranes13010017

**Published:** 2022-12-22

**Authors:** Antara Mazumder, Jung Min Kim, Brock Hunter, Bryan S. Beckingham

**Affiliations:** Department of Chemical Engineering, Auburn University, Auburn, AL 36849, USA

**Keywords:** permeability, multi-component transport, in situ ATR FTIR spectroscopy, ion exchange membrane, PEGDA

## Abstract

Understanding multi-component transport through polymer membranes is critical for separation applications such as water purification, energy devices, etc. Specifically for CO_2_ reduction cells, where the CO_2_ reduction products (alcohols and carboxylate salts), crossover of these species is undesirable and improving the design of ion exchange membranes to prevent this behavior is needed. Previously, it was observed that acetate transport increased in copermeation with alcohols for cation exchange membranes consisting of poly(ethylene glycol) diacrylate (PEGDA) and 2-acrylamido-2-methyl-1-propanesulfonic acid (AMPS) and that the inclusion of poly(ethylene glycol) methacrylate (PEGMA) (*n* = 5, *n* represents the number of ethylene oxide repeat units) could suppress this behavior. Here, we further investigate the role of PEGMA in modulating fractional free volume and transport behavior of alcohols and carboxylates. PEGDA-PEGMA membranes of varied membranes are fabricated with both varied pre −polymerization water content at constant PEGMA (*n* = 9) content and varied PEGMA content at two pre −polymerization water contents (20 and 60 wt.% water). Permeability to sodium acetate also decreases in these charge-neutral PEGDA-PEGMA membranes compared to PEGMA-free films. Therefore, incorporation of comonomers such as PEGMA with long side chains may provide a useful membrane chemistry structural motif for preventing undesirable carboxylate crossover in polymer membranes.

## 1. Introduction

CO_2_ emissions have been a major environmental concern over the last 100 years due to the increasing usage of fossil fuels releasing CO_2_ on a timescale not proportional to the rate of absorption from the environment [1]. Hence, researchers are working relentlessly to find an effective solution for mitigating atmospheric CO_2_ and exploring alternative fuel sources to reduce dependence on fossil fuels. The primary strategies to reduce CO_2_ from the environment include CO_2_ capture, CO_2_ storage, and CO_2_ conversion [2]. Among them, CO_2_ conversion through electrochemical reduction is an attractive approach as it converts CO_2_ into molecules such as alcohols, formate [2], etc. which can be used as fuels or industrial chemicals without relying on petroleum-based resources. An electrochemical cell consists of two independent half-cells (anode and cathode) with the CO_2_ reduction reaction and oxygen evolution reaction (OER) each taking place in one of the half-cells separated by a polymer membrane that allows ion transport between the anode and cathode [2]. The electrochemical reduction process is quite challenging since CO_2_ is a thermodynamically stable molecule due to the strong C=O [3]. The electrochemical reduction process follows multi-electron and multi-proton pathways that typically produces a mixture of products, including methanol, ethanol, formate, formic acid, etc., instead of a single specific desired product [2]. These CO_2_ reduction products are generally soluble in the electrolyte such that they can crossover (permeate) through the polymer membrane allowing oxidation at the anode [4] and reducing cell efficiency. However, limited investigative studies have been reported about efficient electro-chemical cell operation overcoming the problem of potential losses due to non-ideal ion transport across the chambers, resistive losses, and product crossover [5]. Here, one significant role is played by ion-exchange membranes that mitigate product (and reactant) crossover while maintaining the desired transport of electrolytes for cell operation. Multiple studies have sought to improve IEMs in terms of increasing membrane’s mechanical strength and ionic conductivity to sustain the operational conditions for different electrochemical cell applications. In a study by Kim et al. (2017), a ternary hybrid membrane consisting sulfonated fluorinated multi-block copolymer (SFMC), sulfonated (poly ether ether ketone) (SPEEK), and 1 or 5 wt.% graphene oxide (GO) was prepared through solution casting approach for PEM fuel cell application [6]. This hybrid membrane showed improvement in dimensional stability, water absorption, ionic conductivity, and ion exchange capacity (IEC) [6]. Especially the incorporation of GO increased the reinforcement both mechanically and thermally by restructuring the bonds of SFMC and SPEEK. In a study by Ebrasu et al. (2019), graphene was incorporated in the commercial cation exchange membranes (CEM) backbone in different loadings for water electrolysis, and it showed an improvement in proton conduction compared to the pristine commercial membranes [7]. Consequentially, in another study by Ebrasu et al. (2020), a composite graphene-modified anion exchange membrane (AEIM) has been introduced that helps to improve OH^-^ transport through graphene concentration. However, excessive graphene concentration creates a hindrance in OH^−^ ion transport through the conduction channels [8]. A review by Carmo et al. (2013) discussed the development of proton exchange membranes (PEM) in water electrolysis where there was mention of using polybenzimidazoles (PBI), poly(ether ether ketones) (PEEK), poly(ether sulfones) (PES), and sulfonated polyphenyl quinoxaline (SPPQ) in PEM electrolysis by performing sulfonation into ionomers/membranes; Yet, these alternative membranes show low current densities and low durability [9]. Nevertheless, the investigation of CO_2_ reduction products (liquid products) crossover through IEMs has not been extensively investigated, in particular for mixtures of products. Therefore, this study seeks to expand upon prior work investigating transport and cotransport in crosslinked, dense polymer membranes and as such will be also useful to applications where this class of IEMs are such as electrodialysis [10], energy storage devices [11,12], artificial photosynthesis devices [4,13], and wastewater purification [14].

Ion-conducting membranes, such as Nafion™, with robust physical and transport properties and commercial availability, have played a vital role in ion transport applications. However, due to the exclusive nature of these commercially available membranes, it is not easy to ascertain the impact of membrane chemistry and structure in water, electrolyte, and ion transport. To understand how membrane chemistry and structure impact solute transport and co-transport in an electrochemical cell, a tunable membrane platform is required to investigate structure-property relationships systematically. Crosslinked polyether-acrylate-based materials are one such platform that provides the leverage to synthesize membranes of various physiochemical properties by tuning the chemistry and structure [15,16,17,18,19].

Numerous studies [20,21,22,23] have investigated single solute transport for different polymer membrane chemistries to understand transport relationships, such as water and salt transport studies through zwitterionic polymer films [20] or AMPS/PEGDA films [21]. In the work of Freeman and coworkers, it was observed that water, sodium chloride [22], and methanol permeability [23] towards crosslinked poly(ethylene glycol) diacrylate (PEGDA) membranes increase with increasing pre −polymerization water content and comonomer content; as both impact membrane fractional free volume. However, multi-component transport is still at nascent stage compared to the investigation of single-component transport through different membrane structures. In a study by Beckingham al. (2018) [24], the permeability of Nafion to methanol (MeOH) and sodium acetate (NaOAc) was characterized for single solute (1 M in water) and the binary mixture (1 M each in water) where it was observed that Nafion’s relative permeability to MeOH and NaOAc changed for the mixtures compared to expectations from single-solute transport experiments (e.g., permselectivity). Similar behavior was observed by Kim et al. (2020) [16] investigating permeability to methanol and sodium acetate for UV-crosslinked cation exchange membranes (CEMs) synthesized with a sulfonated monomer, 2-acrylamide-2-methylpropane sulfonic acid (AMPS), and PEGDA, (*n* = 13, *n* represents the number of ethylene oxide repeat units), where it was found that permeability to sodium acetate increased while co-permeating with methanol compared to single permeation. From prior studies, it is known that crosslinked PEGDA membranes’ fractional free volume (FFV) can be varied by adding a side chain comonomer such as poly(ethylene glycol) acrylate (PEGA) (*n* = 7) [25], or sulfobetaine methacrylate (SBMA) [26], and the impact on single solute transport properties investigated. This has prompted the investigation of the role of different side chain comonomers on multi-solute transport through crosslinked membranes. Kim et al. (2021) [17] investigated UV-crosslinked PEGDA membranes with three different uncharged comonomers acrylic acid (AA, *n* = 0), hydroxyethyl methacrylate (HEMA, *n* = 1), and poly(ethylene glycol) methacrylate (PEGMA, *n* = 5), where the permeability to carboxylates (including sodium acetate) was suppressed in PEGMA-containing cation exchange membranes in co-transport compared to the other comonomer membranes. To expand understanding of the role of the long-chain pendant group PEGMA in solute transport and co-transport in the PEGDA-based cation exchange membranes, Kim et al. (2022) [18] prepared a series of PEGMA (*n =* 5) containing films with varied PEGMA and AMPS content where permeability to NaOAc decreased in PEGMA containing membranes while co-transporting with MeOH compared to PEGMA-free membranes. 

**Figure 1:**
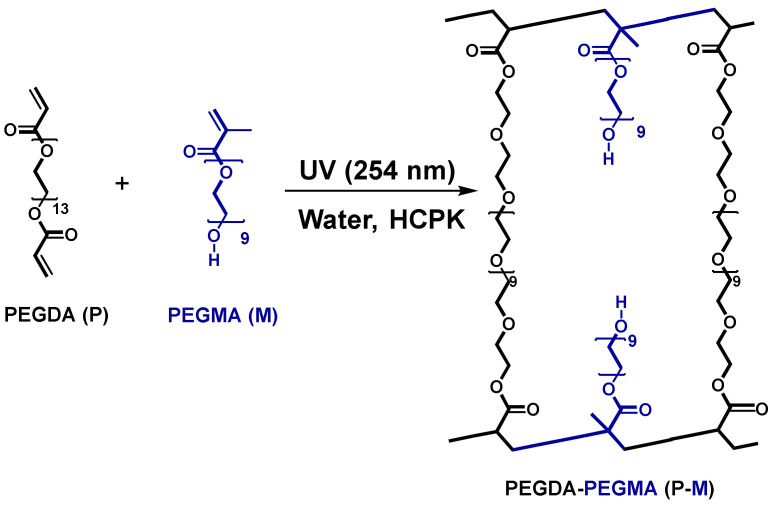
Synthesis of PEGDA-PEGMA membrane.

However, these prior works did not explore the relative impacts of PEGMA and pre −polymerization water content on manipulating physiochemical properties such as variation in the number and size of the fractional free volume (FFV) and its correlation with the single and multi-solute transport. Here, this work aims to fill this gap by systematically investigating the role of PEGMA (*n =* 9) of longer chain length in controlling the fractional free volume (FFV) of charge-neutral membranes and its impact on mechanical and transport properties. Two series of crosslinked PEGDA membranes are synthesized following the scheme described in Figure 1 by varying FFV in two different ways: (1) varying pre−polymerization water content at constant PEGMA content, and (2) varying PEGMA content at constant pre−polymerization water content (as PEGMA also contributes to the FFV in polymer membrane).

## 2. Materials and Methods

### 2.1. Materials, Film Formation, and Physiochemical Parameters Measurement

Poly(ethylene glycol) diacrylate (PEGDA, *n* = 13, Mn = 700) was purchased from Sigma-Aldrich Chemicals (St. Louis, MS, USA) and poly(ethylene glycol) methacrylate (PEGMA, *n* = 9, Mn = 500) was purchased from Polysciences Inc. (Warrington, PA, USA). 1-Hydroxyl-cyclohexyl phenyl ketone (HCPK, photoinitiator) was purchased from Tokyo Chemical Industry (Tokyo, Japan). Methanol (99.8%) and ethanol were purchased from British Drug House (BDH^®^) Chemicals (Poole, UK), and sodium acetate and sodium formate were purchased from ACS Chemical Inc. (Point Pleasant, NJ, USA). Type-1 deionized water produced by a Waterpro BT Purification System from Labconco^®^ (18.2 mΩ cm at 25 °C, 1.2 ppb TOC) (Kansas City, MO, USA) has been used in this work. Polymer films were prepared by UV photopolymerization of pre −polymerization mixtures containing, PEGDA, PEGMA, water, and photoinitiator between quartz plates separated using spacers by 305 μm. The complete experimental details for film formation, and physiochemical property characterization, including water uptake, water volume fraction, dimensional swelling, glass transition temperature (*T_g_*), and storage modulus (*E’*) are provided in the Supporting Materials.

### 2.2. Diffusion Cell Experiments

**Figure 2:**
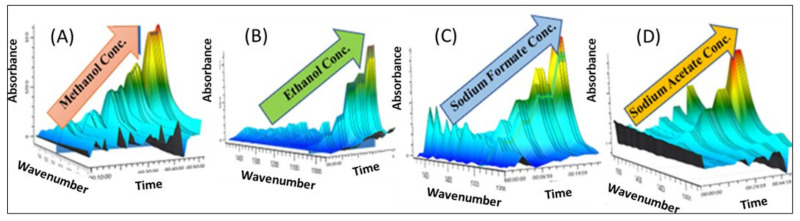
ATR-FTIR spectra at increasing concentrations (**A**) methanol, 1018 cm^−1^, (**B**) ethanol, 1044 cm^−1^, (**C**) sodium formate 1350 cm^−1^, and (**D**) sodium acetate 1414 cm^−1^. Spectra are zoomed in for clarity.

For measuring diffusive permeabilities of neutral solutes (methanol & ethanol) in a single and binary solute transport, a custom-built diffusion cell was used; see Supporting Materials Figure S1. Each half-cell has a 1.1423 cm^2^ orifice with a vertical ground glass face and is jacketed to maintain a temperature of 25 °C throughout. For measuring the diffusive permeability of single-charged solutes (1 M sodium formate or sodium acetate in the donor cell), an in situ conductivity meter was used to monitor the receiver cell concentration via the measured change in solution conductivity determined via calibration of known concentration solutions (Figure 2).

For all diffusion cell experiments containing one of the alcohols (methanol & ethanol), an in situ ATR-FTIR spectroscopy probe (Mettler-Toledo ReactIR™ 15 with shallow tip 9.5 mm DSub AgX DiComp probe, Columbus, OH, USA) was inserted into the sample port of the receiver cell in lieu of the conductivity meter as these solutes do not result in a large enough change in conductivity for accurate measurement. For the ATR FTIR spectroscopy analysis, the molar absorptivity coefficient is obtained following a calibration procedure which has been elaborately described in our previous work [15,16,17,18]; see Supporting Materials Table S2 for molar absorptivity values for all the solutes in all the wavelengths. The concentration versus time data extracted from either the conductivity or absorbance was analyzed by best fitting with the Yasuda model, Equation (1), to extract the permeability values of each solute in single, and multi-solute solutions.

(1) $$\;\;\;\;P_{i}=ln\left(1-\frac{2c_{i,l}\left(t\right)}{c_{i,0}}\right)\left(\frac{-IV}{2At}\right)$$

where *P_i_* (cm^2^/s)is the membrane permeability to solute *i*, *c_i,l_* (*t*) (mol/s) is the concentration of solute *i* in the receiver cell at time *t*, *c_i,_*_0_ (mol/L) is the initial concentration of solute *i* in feed cell (1 M), *l* (cm) is the membrane thickness, and *V* (mL)is the half-cell volume (25 mL).

**Figure 3:**
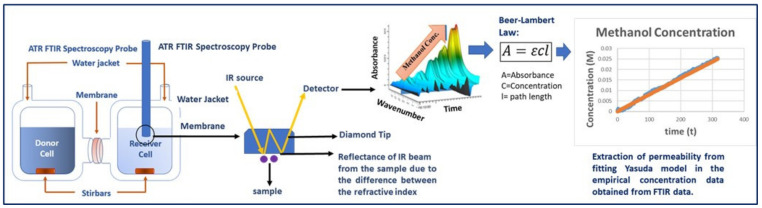
Schematic of multicomponent permeability measurement through the PEGDA-PEGMA membrane using a diffusion cell coupled with in situ ATR-FTIR spectroscopy [27]. Adapted in part from Ref. [27].

The detailed procedure for measuring multi-component permeability utilizing the diffusion cell set up coupled with ATR FTIR spectroscopy has been described in Figure 3. 

## 3. Results

### 3.1. Membrane Synthesis, Water Uptake, Water Volume Fraction, and Crosslinking Density

**Figure 4:**
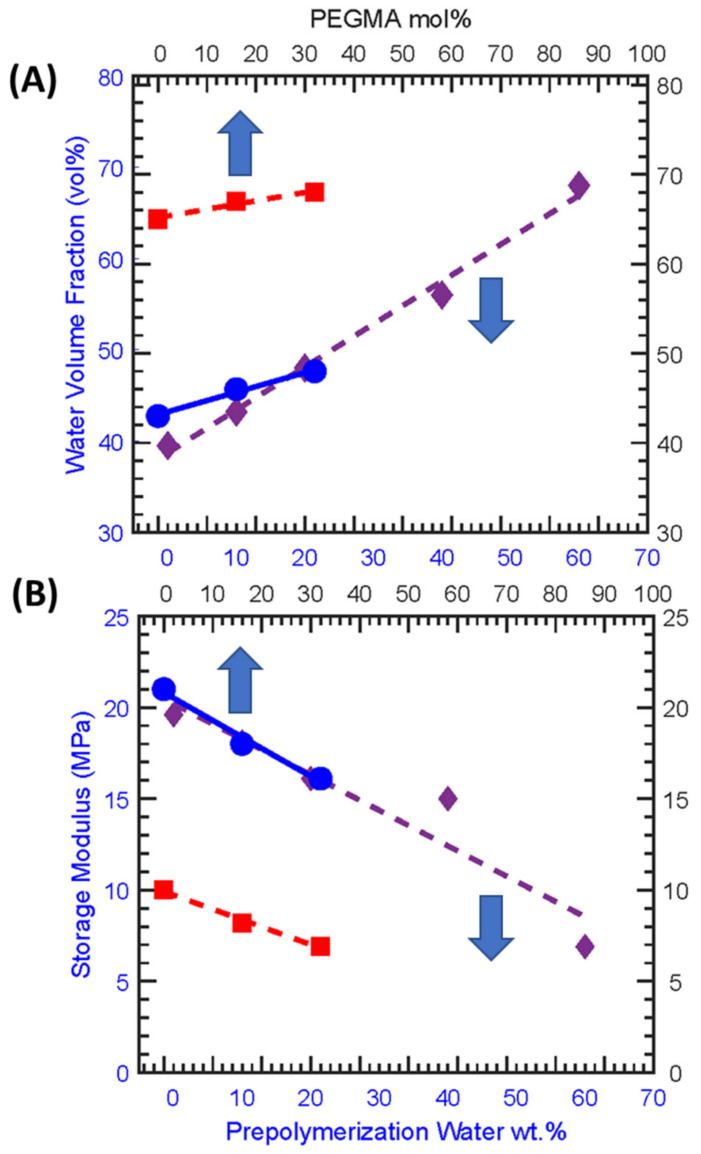
(**A**) Water volume fractions, and (**B**) crosslinking density for polymer films at 0, 10, 20, 40, and 60 wt% pre −polymerization water content, prepared with 32 mol% PEGMA (◆, dash), water volume fractions of films at 0, 16, and 32 mol% (M) PEGMA content, prepared with 20 (●, solid) and 60 wt% (■, dash) pre −polymerization water content. [the upper arrow indicates PEGMA mol% as an x-axis variable, and the lower arrow indicates pre −polymerization water wt.% as an x-axis variable].

PEGDA is a hydrophilic polyether with two acrylate chain ends that facilitate crosslinking upon free radical polymerization such as via a photoinitiator (HCPK here) and UV light as performed here. Two series of freestanding crosslinked PEGDA membranes were prepared (1) constant PEGMA (M, uncharged comonomer) content and varied solvent (water) content in the pre−polymerization solution and (2) varied PEGMA content in the pre−polymerization solution at fixed pre−polymerization water content, mixture compositions are given in Supporting Materials Table S1. Here, we denote these membranes as XY−MYY where XY indicates the pre−polymerization water content and YY indicates PEGMA content. Previously, it has been observed that increasing water content [15] or increasing the comonomer content in the pre−polymerization mixture leads to higher FFV in the synthesized membranes [17,22]. To assess the relative amount of FFV in membranes, equilibrium water uptake and water volume fraction are characterized. Water uptake and water volume fraction are commonly utilized as estimates for relative FFV as water molecules occupy the available fractional free volume in the dense, hydrated polymeric membranes upon hydration. Understanding the relative FFV content in the membrane is important for understanding transport behavior as FFV relates directly to solute uptake and solute diffusion behavior through the membrane [21,22]. In both series of synthesized membranes, the equilibrium water content and water volume fraction linearly increase with increasing water or PEGMA content in the pre−polymerization mixture as shown in Figure 4 (see Supporting Materials Table S3 for the values). This increasing water uptake and water volume fraction with increasing pre−polymerization water or PEGMA content are consistent with an increasing amount of FFV in the membrane. This phenomenon is attributed to the decreasing crosslinking density upon adding comonomer or increasing water content in the pre−polymerization mixture as both result in a more open network structure and ultimately additional FFV within the film. It is clear from the relative range of water volume observed in Figure 4 that PEGMA content has an overall weaker impact on FFV than pre−polymerization water content, which is consistent with the relative expected impacts of these two variations on the polymer membrane network structure.

Crosslinking density for the synthesized membrane was estimated from the mechanical properties. The shear modulus (*G,* MPa) of all the synthesized membranes at the rubbery plateau above *T_g_* was measured by performing dynamic mechanical testing (Supporting Materials Figure S3) and the storage modulus (*E,* MPa) (see Supporting Materials Table S3 for the values) was calculated following Equation (2) and crosslinking density ($v_{e}$, mol/cm^3^) calculated from Equation (3) where, *R* is the universal molar gas constant (8.314 J/mol·K), and T (K) is the temperature. Here, the shear modulus and storage modulus at the rubbery plateau are considered at 30 °C above the glass transition temperature (*T_g_*).

*E* = 2*G*(1 + *v*) (2)

The poisson ratio (*v*) for PEGDA is assumed as 0.5 due to its elastomeric character [28].

(3) $$\;v_{e}=\frac{E^{\prime }}{3RT}$$

The storage modulus and effective crosslinking density (see Supporting Materials Table S3 for values and Figure 4B) decrease with the decreasing crosslinker content, increasing pre−polymerization water content, or increasing PEGMA content. A sharp decrease in storage modulus is observed for 60 wt.% pre−polymerization water and 32 mol% PEGMA content, whereas for the addition of PEGMA the drop in storage modulus is less significant (see Supporting Materials Figure S2). This result matches well with water uptake and water volume fraction data where PEGMA has a smaller contribution in increasing PEGDA membrane’s FFV and is consistent with the amount of FFV proportionally affecting the storage modulus and crosslinking density.

### 3.2. Glass Transition Temperature 

**Figure 5:**
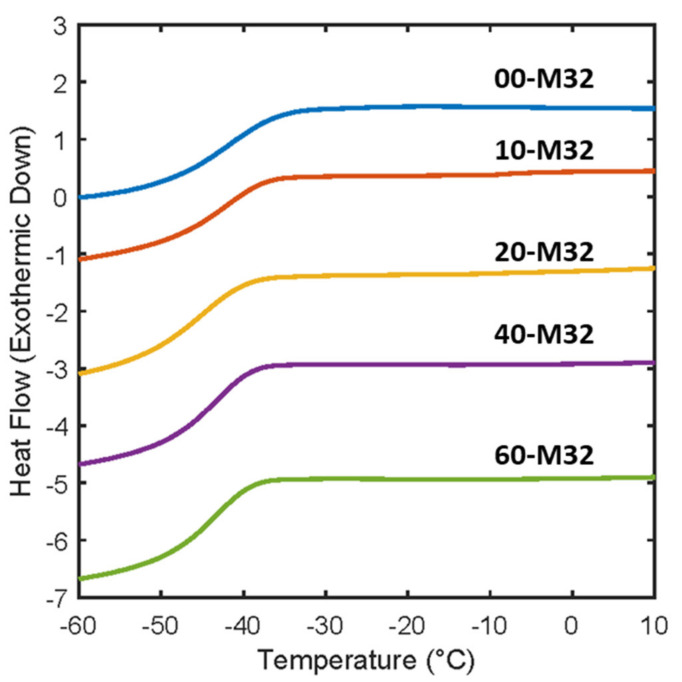
Exemplary differential scanning calorimetry thermograms of PEGDA films with varying pre-polymerization water content at constant PEGMA.

The *T_g_* is an important physical property for understanding relative transport behavior, as it is an indication of polymer chain mobility which impacts the ability of solutes to migrate between FFV elements. The *T_g_* of the synthesized membranes was extracted from the DMA results and determined using differential scanning calorimetry (DSC). Exemplary DSC thermograms for membranes synthesized with varied pre −polymerization water content are given in Figure 5 and all the determined *T_g_*s are provided in the Supporting Materials Table S4. Here, all *T_g_*s fall within a narrow range (−41.2 °C ± 1.6 °C), with no clear differences observed due to the changes in effective crosslink density. Similar behavior has been observed in separate studies by Sumod et al. [25] and Lin et al. [29]. As neither varying water content nor PEGMA comonomer content has any notable effect on the *T_g_* of the dry PEGDA membrane, the impact of structural changes due to either varied composition (PEGMA content) or pre −polymerization water content on segmental relaxation of the films may also be negligible. However, further investigation is needed to confirm similar behavior within hydrated films and thereby assessment of the impact of structure on segmental relaxation and, ultimately, transport behavior.

### 3.3. Dimensional Swelling

**Figure 6:**
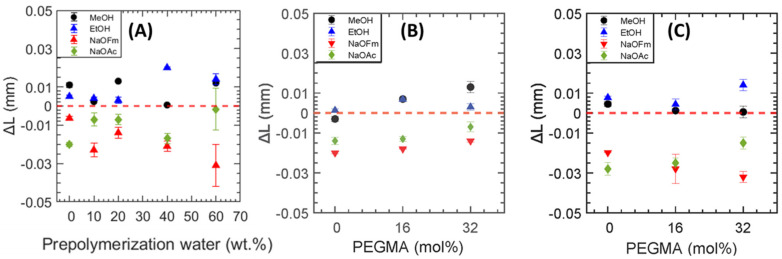
Dimensional Swelling as membrane thickness (Δ*L*, mm) as a function of (**A**) pre−polymerization water wt% at 32 mol% PEGMA, (**B**) varying PEGMA content at 20 wt% pre−polymerization water content, and (**C**) varying PEGMA content at 60 wt% pre−polymerization water content PEGDA based films.

Dimensional swelling (Δ*L*, mm) for all the hydrated PEGDA-based films was characterized after equilibration with a 1 M solution of interest including (1 M methanol (MeOH), 1 M ethanol (EtOH), 1 M sodium formate (NaOFm), 1 M sodium acetate (NaOAc), and 1 M solutions containing equivalent amount of each alcohol and carboxylate salt) using Equation (4).

(4) $$\Delta L=L_{s}-L_{w}$$

where *L_S_* (mm) is the membrane thickness after solution uptake equilibration and *L_W_* (mm) is the membrane thickness after equilibration with water. The relative dimensional swelling/deswelling for all the films is visually represented in Figure 6 where the red dotted line represents no change in membrane thickness after equilibration in the solution of interest. After equilibration with 1 M MeOH and 1 M EtOH, the films (varying pre-polymerization water or varying PEGMA mol%) tend to swell. In contrast, deswelling is observed after equilibration with 1 M NaOFm and NaOAc. Analogous behavior was previously observed for MeOH and NaOAc by Beckingham et al. [24] in Nafion™ 117 and by Dobyns et al. [15] in PEGDA-based films prepared by varying pre −polymerization water content; among others. This behavior has been attributed to the relatively high uptake of alcohols in the membrane leading to swelling [30], whereas the carboxylate salts result in osmotic deswelling [31].

### 3.4. Single and Multi-Solute Permeability

#### 3.4.1. Single Solute Permeability

To investigate the impact of varying fractional free volume by these two routes on transport, membranes of varied fractional free volume were synthesized and their permeability to two alcohols (MeOH and EtOH) and two carboxylate ions (NaOFm and NaOAc) were investigated. The diffusive permeabilities of all the solutes (alcohols and carboxylates) were measured in at least triplicate leveraging either in situ Attenuated Total Reflectance (ATR) FTIR spectroscopy or in situ conductivity probes, as appropriate, with their permeability extracted from the obtained concentration versus time data by fitting with the Yasuda model (Equation (1)) [32]; all permeability values are provided in Supporting Materials Tables S5–S8.

**Figure 7:**
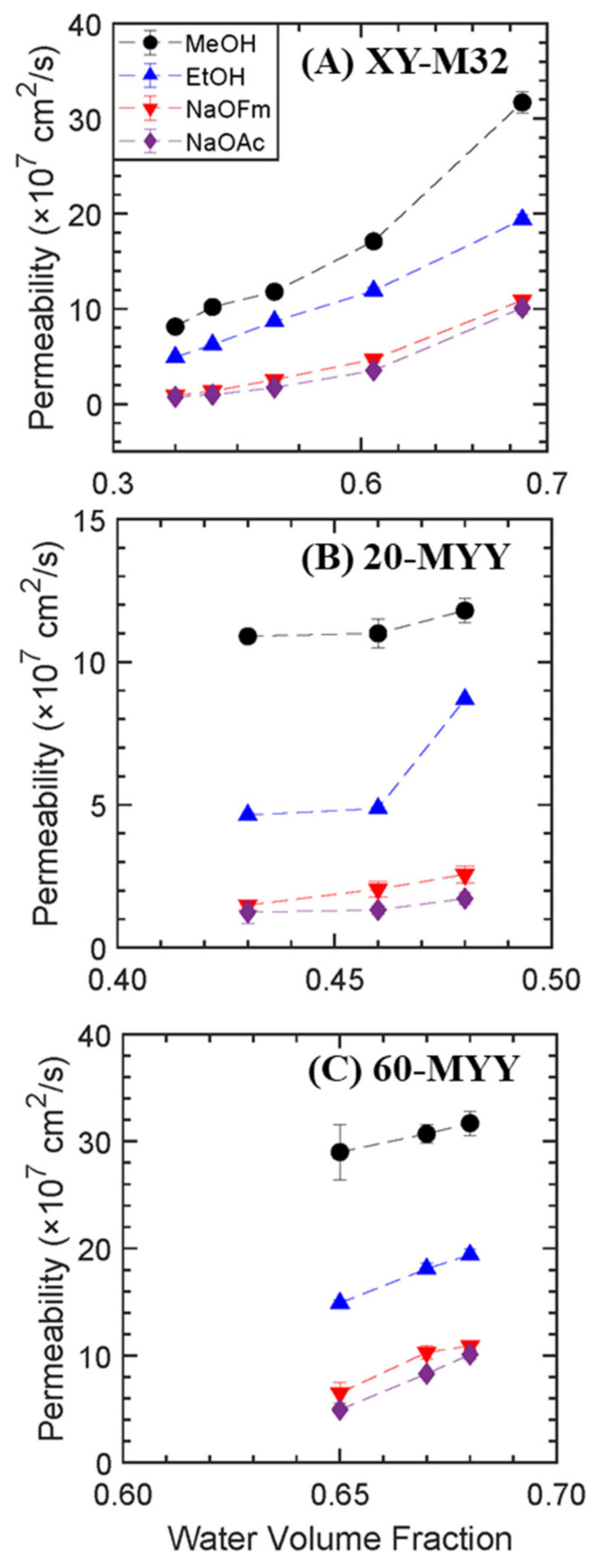
PEGDA-PEGMA permeability to MeOH (●), EtOH (▲), NaOFm (▼), and NaOAc (◆) in single solute solution vs. water volume fraction (**A**) at varied pre −polymerization water content and constant 32 mol% PEGMA content (XY−M32) (**B**) at varied PEGMA content with 20 wt.% pre −polymerization water content (20−MYY), and (**C**) at varied PEGMA content with 60 wt.% pre −polymerization water content (60−MYY).

The single-component permeability for all the solutes exponentially increases with increasing water volume fraction where the pre −polymerization water content has been varied at constant 32 mol% PEGMA content as shown in Figure 7A. According to Yasuda et al. [23,32], solute transport is governed by the free volume theory of diffusion and the solute diffusion coefficient exponentially varies with the reciprocal of free volume. A further assumption made by Yasuda et al. [22] is that the free volume within the membrane is proportional to the equilibrium water uptake, as the diffusion of solutes through a dry membrane is presumed to be negligible. Hence, solute migration occurs through the fractional free volume effectively provided by the water in the hydrogel [23]. Thus, the increasing trend of the solute permeability with the amount of pre−polymerization water is reasonable as it mirrors the observed increase of equilibrium water content and water volume fraction with increasing pre −polymerization water content. 

The diffusive permeability of the single solutes also increases with increasing PEGMA content. Adding PEGMA into the structure increases the fractional free volume (as determined by water uptake) and decreases the crosslinking density; Figure 7. This finding is in good agreement with the work of Freeman and coworkers [22,23], who observed that increasing both pre −polymerization water content and comonomer content led to increasing water, sodium chloride [22], and MeOH [23] permeability. The larger accessible variation in pre−polymerization water content leads to a much larger variation in water volume fraction and, thereby, permeability. 

Looking at the relative solute permeabilities, MeOH permeability is 1.6 times higher on average than the EtOH permeability and NaOFm permeability is 1.3 times higher than NaOAc permeability on average. These relative permeabilities are attributed to the differences in solute size, kinetic diameter for the alcohols, and hydrated diameter for the carboxylate salts. The kinetic diameter of EtOH (4.5 Å [33]) is higher than the kinetic diameter of MeOH (3.6 Å [34]), and the hydrated diameter of OFm^−^ (5.9 Å [35]) is smaller than the hydrated diameter of OAc^−^ (7.44 Å [32,36]). The smaller solutes permeate faster than the larger solutes (diffusivity varies inversely proportional to the solute size).

#### 3.4.2. Multi-Solute Permeability

As the presence of a cosolute can impact permeation behavior, the permeability of each solute for binary mixtures of each combination of alcohol with carboxylate was characterized. In this co-permeation study of alcohols and carboxylate ions, divergent transport behavior was observed from single to co-transport (see Supporting Materials Tables S5–S8).

**Figure 8:**
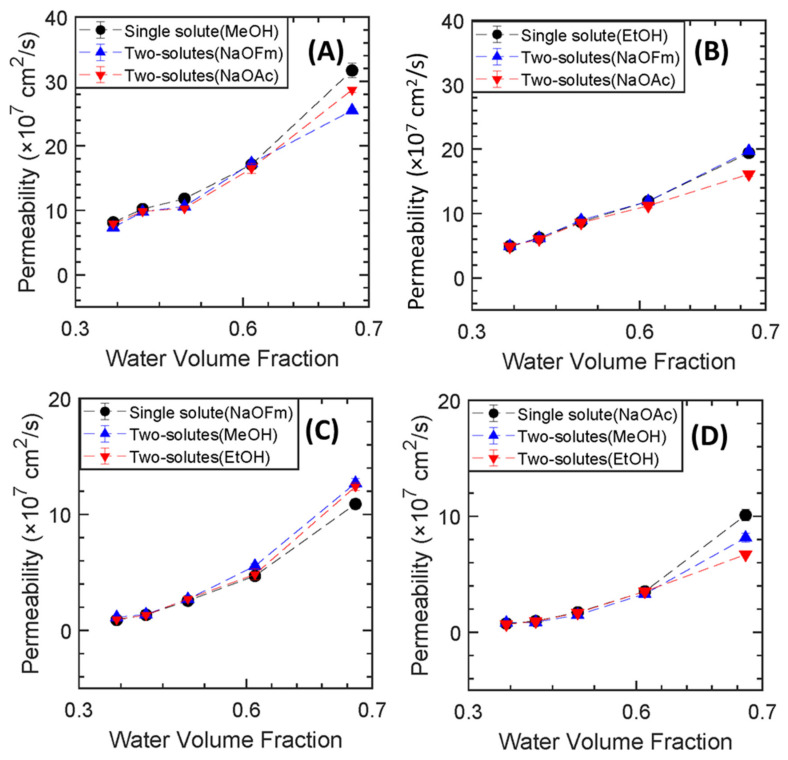
PEGDA-PEGMA permeability to (**A**) MeOH, (**B**) EtOH, (**C**) NaOFm, and (**D**) NaOAc in single and binary mixtures vs. water volume fraction for varied pre−polymerization water content at 32 mol% PEGMA.

First, the transport behavior of alcohols and carboxylate ions in co-permeation will be discussed for membranes with varied pre−polymerization water content at a constant PEGMA mol% (0, 10, 20, 40 and 60−M32) (Figure 8). In co-permeation with NaOFm or NaOAc, MeOH permeability through all the synthesized PEGDA-PEGMA membranes at varied pre −polymerization water content decreased, though to varying amounts. This behavior is presumably due to the competitive diffusion of MeOH with a slower-diffusing molecule that obstructs MeOH transport through the fractional free volume compared to methanol diffusing alone [18]. In the study by Kim et al. (2022), nine series of PEGDA membranes consisting of varying amounts of PEGMA and AMPS were prepared, and for all the membranes, methanol’s permeability was found to be lower in co-transport [18]. EtOH permeability remains essentially the same in all the films at varied pre −polymerization water content with both the NaOFm and NaOAc. However, at higher pre−polymerization water content, and thereby higher water volume fraction, EtOH permeability decreases in co-permeation with NaOAc. Recently, in a study by Kim et al. ***(2021)***, it was observed that EtOH permeability remained essentially the same in PEGDA-based films in single and co-transport with carboxylate ions, which was attributed to the larger observed differences in sorption behavior of EtOH for these mixtures (ethanol’s solubility was 0.298 whereas the solubility was 0.386 while co-transporting with NaOFm) [34]. Thus, in our current study, the consistency in permeability values for EtOH between single and co-permeation in PEGDA-PEGMA films with NaOFm is likely also a result of analogous sorption differences that might compensate for the difference in the diffusive behavior between two molecules. For the carboxylate salts, the permeability of NaOFm in co-permeation with MeOH and EtOH increases in all the PEGDA-PEGMA films at varied pre−polymerization water content (Figure 8C). Conversely, the permeability to NaOAc decreases in co-transport with MeOH and EtOH (Figure 8D). As with MeOH and EtOH, the observed differences are smaller in absolute terms at lower water volume fraction, with the largest differences observed at the highest water volume fraction. While the underlying reason for this difference is unclear, two contributing factors are likely to be differences in sorption behavior as well as the differences in hydrated radius (NaOAc has a larger hydrated radius than NaOFm) [34].

**Figure 9:**
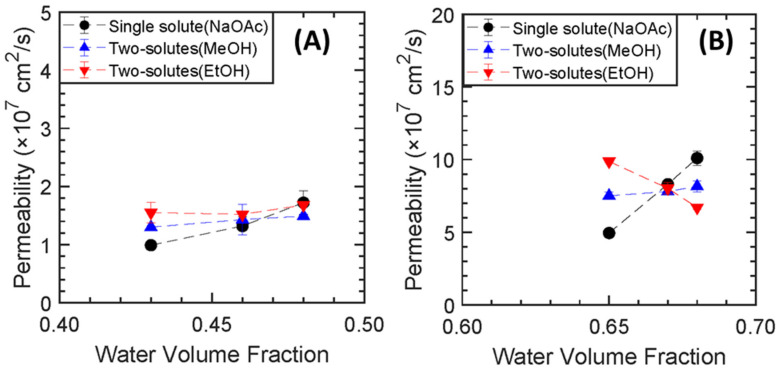
PEGDA-PEGMA (0, 16, 32 mol%) permeability to sodium acetate (NaOAc), in single and two-solute vs. varied PEGMA mol% at (**A**) 20% and (**B**) 60 pre−polymerization water wt%.

For the membranes where the fractional free volume is varied by PEGMA content (from 0 to 16 mol%) at two different pre−polymerization content (20 and 60 wt.%) the differences in transport behavior due to primarily the presence of PEGMA is investigated (see all the permeability values in Supporting Materials Table S5–S8). From Figure 9, the presence of PEGMA clearly impacts solute transport, and in disparate ways depending on solute and water volume fraction. Although the presence of a side chain comonomer PEGMA impacts the solutes and co-solutes in disparate ways, the most interesting emergent transport behavior is observed for the carboxylate salt sodium acetate (NaOAc). In co-permeation, permeability to NaOAc increases with MeOH in PEGMA-free films prepared with both 20 and 60 wt.% pre−polymerization water content. Conversely, in PEGMA-containing films, NaOAc permeability decreases during co-transport with MeOH. This behavior agrees with a previous study by Kim et al. [18] where it was observed that NaOAc transport was suppressed while co-transporting with MeOH in PEGDA-PEGMA-AMPS films. With EtOH, NaOAc permeability is slightly reduced for the PEGMA-containing film compared to the PEGMA-free film at 20 wt.% water whereas the reduction factor is higher in PEGMA-containing film at 60 wt.% pre −polymerization water content. This transport behavior implies the presence of a side chain comonomer PEGMA impedes acetate crossover even in charge-neutral membranes.

In an investigation by Ju et al. (2010) [22], PALS measurements found that films prepared with increasing PEGMA content generally have smaller free-volume elements. Additionally, among the four solutes investigated in the study, NaOAc is the largest (largest effective diameter). Thus, this relative sizing between FFV at varied PEGMA content and solute size may be one contributing factor to the emergent transport behavior (even at increasing water content) and especially so for NaOAC. We have also observed the different co-transport behavior of MeOH, EtOH, and NaOFm for the membranes with varying PEGMA content (20−M00,20−M16, 20−M32, and 60−M00, 60−M16 and 60−M32) (in Supporting Materials Figures S4A–C and S5A–C). MeOH permeability to all the films generally decreases while co-transporting with both NaOFm and NaOAc (Figures S4A and S5A). This behavior is ascribed to the competitive transport of MeOH with slow-moving carboxylate ions [18]. Another interesting phenomenon was seen for the EtOH while co-transporting with NaOAc. Although EtOH’s permeability increases in PEGMA-free films in co-transport with NaOAc, it decreases in the PEGMA-containing film at higher water volume fraction (Figure 8 and Figure S5B). This may be due to the impact of PEGMA on the transport of the large and slow diffusing NaOAc which then impacts the EtOH. NaOFm show slightly different behavior compared to NaOAc in co-transport for the membranes at varied PEGMA mol%. In co-permeation, NaOFm permeability increased in all films varying PEGMA content with both MeOH and EtOH except in the films containing 16 mol% PEGMA where the permeabilities remain almost the same. The reason for the increasing permeability of NaOFm in co-transport with alcohols may be due to a larger relative impact of the fast-diffusing alcohols on NaOFm as it is smaller in size than NaOAc.

## 4. Conclusions

This study explicitly investigated and compared the relative contributions of pre−polymerization water content and PEGMA content in altering membrane physiochemical properties and its eventual effect on solute transport behavior. Two series of membranes were prepared: (1) by varying pre −polymerization water content at a constant PEGMA mol% and (2) by varying PEGMA content at two constant pre−polymerization water content (00−M32, 10−M32, 20−M32, 40−M32, 60−M32). To characterize the prepared membranes, important physiochemical properties and transport behavior were measured and discussed. Both pre −polymerization water content and PEGMA content are methodologies for tuning the water volume fraction in polymer membranes, with pre−polymerization water content having a larger impact than PEGMA. The permeability of the single solutes increased with the increasing pre−polymerization of water content and PEGMA content due to higher water volume fraction and fractional free volume. However, distinct behavior is observed for the alcohols and carboxylates in co-transport. This distinct behavior is more significant at higher water volume fractions, is complex, and varies by solute/co-solute pair. Notably, NaOAc permeability increased in PEGMA-free films during co-transport with alcohols and decreased in PEGMA-containing films. The hypothesis about this behavior is related to NaOFm’s higher solubility than NaOAc and the presence of the PEGMA chain in the PEGDA films. Overall, it can be said that the permeability of a solute is not only influenced by the membrane FFV (as measured by relative water uptake) but also by solute-solute and solute-membrane interactions during transport. In the future, we aim to further investigate this complex and interesting phenomena in the case of charged membranes as much remains unknown about the array of complex interactions that lead to this behavior and a greater understanding may lead to insights for the design of improved membranes for electrochemical devices which allow for device operation but prevents the undesired crossover of species (such as MeOH, EtOH, NaOFM, and NaOAc in solar fuels devices). Here, the use of PEGMA as a neutral comonomer which leads to depressed transport of carboxylates in co-transport with alcohols is both interesting and potentially a strategy for improving IEMs for CO_2_ reduction devices.

## Data Availability

The data presented in this study are contained herein and in the supporting information. Any additional data is available upon reasonable request.

## Supplementary Materials

The following supporting information can be downloaded at: https://www.mdpi.com/article/10.3390/membranes13010017/s1, additional experimental details; Table S1: Membrane pre −polymerization mixture formulations; Table S2: Molar absorptivity of methanol (MeOH), ethanol (EtOH), sodium formate (NaOFm) and sodium acetate (NaOAc) extracted from calibration solutions; Table S3: Water uptake, water volume fraction, storage modulus, and crosslinking density for the range of PEGDA-PEGMA-water membranes; Table S4: Glass transition temperature (T_g_) of all the PEGDA-PEGMA membranes; Table S5: Diffusive permeabilities of PEGDA based films varying pre −polymerization water wt% and PEGMA mol% to MeOH and NaOAc in single and two-solute measurements; Table S6: Diffusive permeabilities of PEGDA based films varying pre−polymerization water wt% and PEGMA mol% to MeOH and NaOFm in single and two-solute measurements; Table S7: Diffusive permeabilities of PEGDA based films varying pre−polymerization water wt% and PEGMA mol% to EtOH and NaOAc in single and two-solute measurements; Table S8: Diffusive permeabilities of PEGDA based films varying pre−polymerization water wt% and PEGMA mol% to EtOH and NaOFm in single and two-solute measurements; Table S9: True and Ideal Selectivity for Methanol (MeOH) and Ethanol (EtOH) over Sodium Formate (NaOFm); Figure S1: A custom-build diffusion cell experiment; Figure S2: Storage modulus measured at rubbery plateau for series of PEGDA membranes varying (**A**) pre−polymerization water content at 32 mol% PEGMA (**B**) PEGMA mol% at 20 wt.% pre−polymerization water content, and (**C**) PEGMA mol% at 60 wt.% pre −polymerization water content; Figure S3: PEGDA-PEGMA (0, 16, 32 mol%) permeability to (**A**) methanol (MeOH), (**B**) ethanol (EtOH) (**C**) sodium formate (NaOFm),in single and two-solute vs. varied PEGMA mol% at 20% pre−polymerization water wt%; Figure S4: PEGDA-PEGMA (0, 16, 32 mol%) permeability to (**A**) methanol (MeOH), (**B**) ethanol (EtOH) (**C**) sodium formate (NaOFm),in single and two-solute vs. varied PEGMA mol% at 60% pre−polymerization water wt%. References [16,17,18,37,38,39] were cited in Supplementary Materials.
