# Supplementary material for: Controlling Fractional Free Volume, Transport, and Co-Transport of Alcohols and Carboxylate Salts in PEGDA Membranes
Source: Membranes (Basel). 2022 Dec 22;13(1):17. doi: 10.3390/membranes13010017 (PMC9862150; doi:10.3390/membranes13010017)
Supplement: Supplementary file 1 [file membranes-13-00017-s001.zip › membranes-2045758-supplementary.pdf]

Supplementary Materials

Controlling Fractional Free Volume, Transport, and Co-Transport of Alcohols and Carboxylate Salts in PEGDA Membranes

Antara Mazumder, Jung Min Kim, Brock Hunter and Bryan S. Beckingham *

Department of Chemical Engineering, Auburn University, Auburn, AL 36849, USA;
* Correspondence: bsb0025@auburn.edu

Experimental Methods:

Film Formation

The UV crosslinking method for preparing PEGDA-PEGMA membranes has been described in some of the previous works [1–3]. PEGDA and PEGDA-PEGMA membranes are synthesized by UV-photopolymerization in the presence of photoinitiator HCPK (1-hydroxycyclohexyl phenyl ketone). In the first series of membranes, the PEGMA and PEGDA content were fixed (32 mol% PEGMA and 68 mol% PEGDA based solely on the PEGDA and PEGMA in the final mixture) and the pre-polymerization water content var-ied from 0 to 60 wt.% (0 to 6 g) (Table S1). For the second series of membranes, the amount of pre-polymerization water is kept constant at either 20 wt.% or 60 wt.% (2 g and 6 g respectively) and the PEGMA/PEGDA content varied for final mixture compositions from 0 to 32 mol% PEGMA and balance PEGDA (similarly based on solely the PEGDA and PEGMA content in the mixture) (Table S1). Here, the highest PEGMA content was selected based on the maximum solubility of PEGMA. The pre-polymerization mixture was then sonicated for at least 30 min to produce a transparent mixture and constrained between two 5×5″ (1/4″ thickness) quartz plates having steel spacers (305 μm) placed on two sides of the solution clipped by 4 binders. These constrained solutions were then placed inside a UV crosslinking oven, Spectrolinker XL-1500 from Spectroline, Melville, NY, USA under 254 nm for 3 min at 3.0 mW/cm^2. After cross-linking the films were removed from the quartz plates and immersed in ultrapure water for at least 48 h to fully hydrate the membrane before measuring physicochemical properties and performing transport experiments.

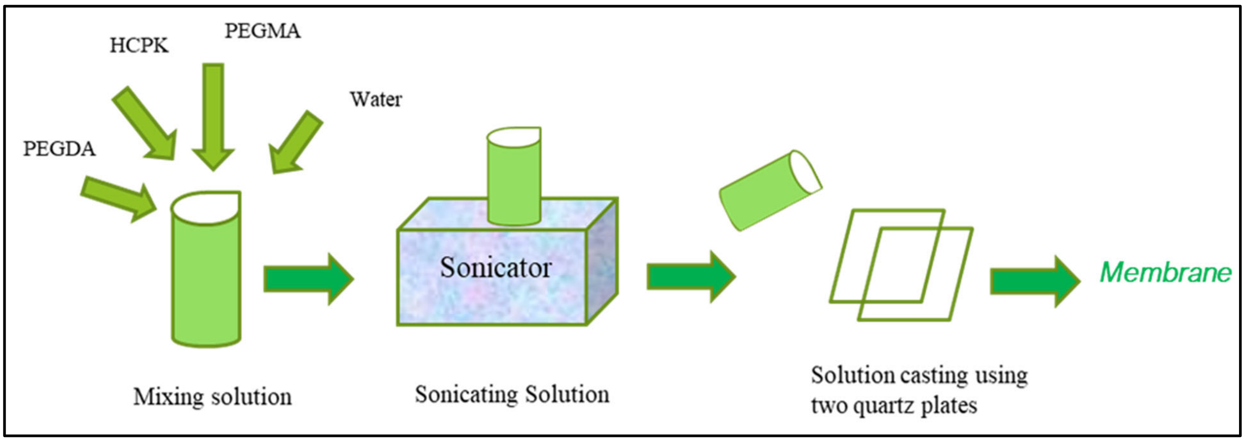

(**a**)

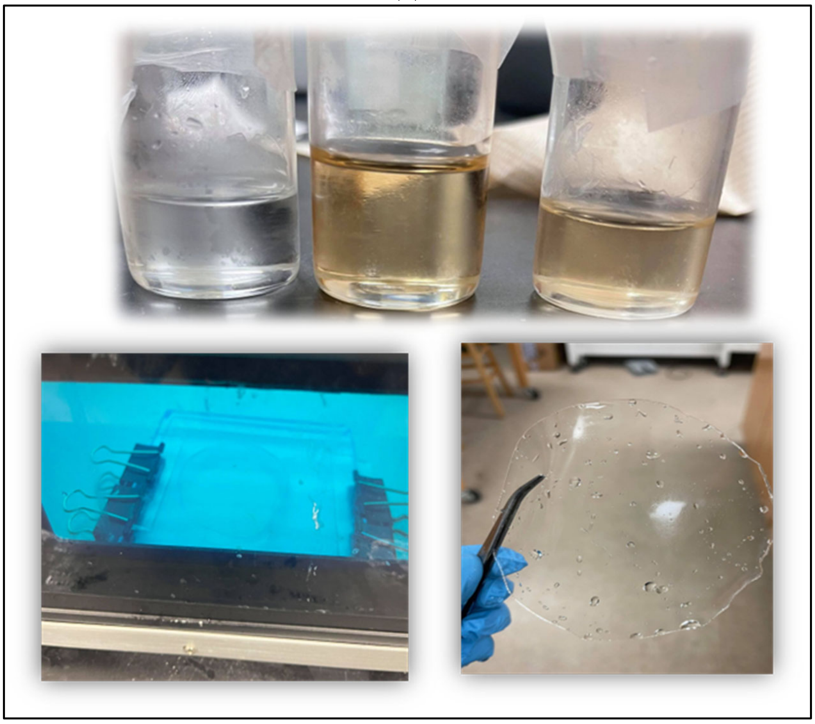

(**b**)

Figure S1. A visual representation of crosslinked membrane synthesis process by UV free radical polymerization. (**a**) schematic diagram of the membrane preparation process and (**b**) photograph of the pre−polymerization solution, crosslinking process occurring inside the UV oven, and the synthesized membranes.

Table S1. Membrane pre − polymerization mixture formulations.

Membrane	Water[a] (wt.%)	PEGMA[b] (mol%)	PEGDA (g)	PEGMA (g)	Water (g)	HCPK (g)
00−M32	0	32	7.484	2.516	0.000	0.010
10−M32	10	32	6.804	2.287	0.909	0.009
20−M32	20	32	5.987	2.013	2.000	0.008
40−M32	40	32	4.491	1.509	4.000	0.006
60−M32	60	32	2.994	1.006	6.000	0.004
20−M00	20	00	8.000	0.000	2.000	0.008
20−M16	20	16	7.042	0.958	2.000	0.008
60−M00	60	00	4.000	0.000	6.000	0.004
60−M16	60	16	3.521	0.479	6.000	0.004

[a] Water = Weight of Water/(Weight of Water + Weight of PEGDA + Weight of PEGMA) × 100%
[b] PEGMA = mol of PEGMA/(mol of PEGDA + mol of PEGMA) × 100%.

Water Content, Water Volume Fraction, Glass Transition Temperature, Storage Modulus and Dimensional Swelling

The detailed experimental procedures have been described in the literature [1–6]. The gravimetric method was used to measure water uptake and the buoyancy method was followed to measure water volume fraction. The mass of all the hydrated PEGDA-PEGMA membranes (0.75-inch diameter hole punch), W_s, was measured after quickly absorbing the surface water using Kimwipes (Kimtech Science™ Kimwipes™ Delicate Task Wipes, Roswell, GA, USA). After that, the membranes were then dried in a vacuum oven (VWR Collection Models 89508-426, Radnor, PA,USA) at 50 °C, and the mass of the dried membrane, W_d, measured. The water uptake, ω_w, for all films was calculated using Equation (S1).

$$\omega_w = \frac{W_s - W_d}{W_d} \quad \text{(S1)}$$

For the calculated water volume fraction, the density of the dry membrane was measured with a density kit coupled with a scale (ML204T, Mettler Toledo, Columbus, OH, USA) at 22 °C. The density, ρ_p, was calculated as Equation (S2).

$$\rho_p = (\rho_L - \rho_0)(\frac{W_0}{W_0 - W_L}) + \rho_0 \quad \text{(S2)}$$

where ρ_L is the density of water (997.8 kg/m^3 at 22 °C), ρ_0 is the density of air (1.225 kg/m^3), W_0 is the weight of the dried film in air, and W_L is the weight of the film in water.

Water volume fraction, ϕ_w, was calculated using Equation (S3)

$$\phi_w = \frac{(W_s - W_d)/\rho_w}{(W_s - W_d)/\rho_w + W_d/\rho_p} \quad \text{(S3)}$$

where ρ_w is the density of the water and ρ_p is the density of the polymer.

For determining dimensional swelling, the thickness of swollen PEGDA-PEGMA membranes was measured after immersing in 1 M methanol, 1 M ethanol, 1 M methanol & acetate, 1 M methanol & formate, 1 M ethanol & acetate and 1 M ethanol & formate for 3 days. Before immersing, a hole punch was used to cut 3 hydrated films of each membrane for each solution. The solution was replaced daily. A digital caliper (±1 μm) was used to measure the thickness, t, of all films at 5 different points.

Storage Modulus

The storage modulus (E') of the synthesized membranes was measured using a torsional fixture with a dynamic mechanical analyzer (DMA, TA instruments DHR20, New

Castle, DE, USA). The thickness of the membrane samples was approximately 1 mm. After preparing the samples, they were hydrated for at least 5 days and then vacuum dried at 50 °C for 24 h. The dried films were cut into rectangular shapes maintaining a dimension of approximately 10×30 mm. The storage modulus of the membranes was then measured with DMA at a heating rate of 5 °C/minute from −60 °C to 30°C at a test frequency of 1 Hz maintaining the liquid nitrogen (N_2) environment.

Glass Transition Temperature

TA instrument (New Castle, DE, USA) Q1000 differential scanning calorimeter (DSC) was used to determine glass transition temperature (T_g). Samples (8–10 mg) were scanned at a heating rate of 10 °C/min from −90 to 50 °C. The glass transition temperature was extracted as the midpoint of the glass transition step. Glass transition temperatures have also been extracted from the highest tanδ values by running the DMA (specification described in the storage modulus section) experiments.

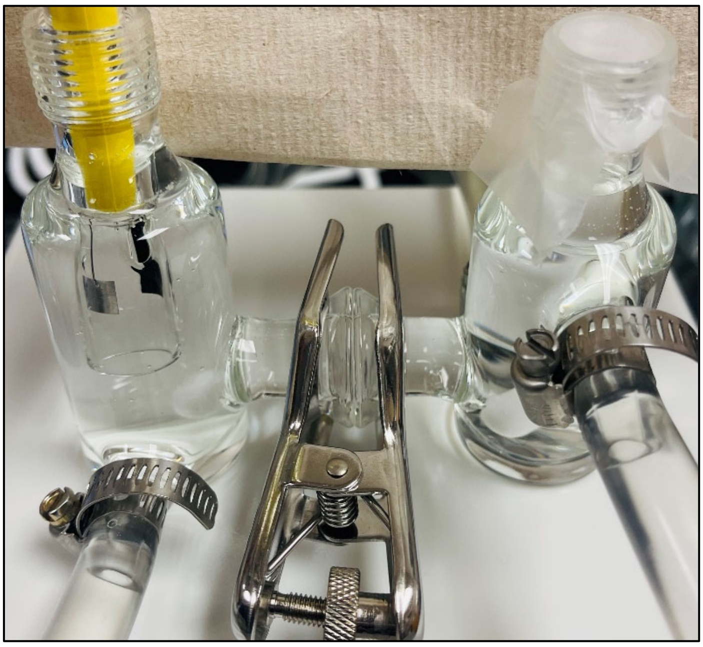

Figure S2. A custom-built diffusion cell experiment.

Table S2. Molar absorptivity of methanol (MeOH), ethanol (EtOH), sodium formate (NaOFm) and sodium acetate (NaOAc) extracted from calibration solutions.

Molar Absorptivity	MeOH	EtOH	NaOFm	NaOAc
ε_{1018}	0.1626	-	−0.014	0.0340
ε_{1044}	-	0.1747	−0.014	0.0344
ε_{1414}	0.0125	0.0181	-	0.2640
ε_{1350}	−0.0004	−0.0002	0.121	-

Table S3. Water uptake, water volume fraction, storage modulus, and crosslinking density for the range of PEGDA-PEGMA-water membranes.

Membrane	Water Uptake (g/g dry membrane)	Water Volume Fraction	Storage Modulus (MPa)	Crosslinking Density (mol/cm^3)
00–M32	0.53 ± 0.009	0.40 ± 0.005	19.6	3.1
10–M32	0.63 ± 0.010	0.43 ± 0.010	18.1	2.7
20–M32	0.74 ± 0.020	0.48 ± 0.007	16.1	2.4
40–M32	1.00 ± 0.040	0.56 ± 0.009	15.5	2.2
60–M32	1.87 ± 0.040	0.68 ± 0.010	6.9	1.0
20–M00	0.64 ± 0.020	0.43 ± 0.007	21.0	3.1
20–M16	0.65 ± 0.020	0.46 ± 0.008	18.0	2.6
60–M00	1.54 ± 0.010	0.65 ± 0.007	10.0	1.4
60–M16	1.69 ± 0.040	0.67 ± 0.006	8.2	1.2

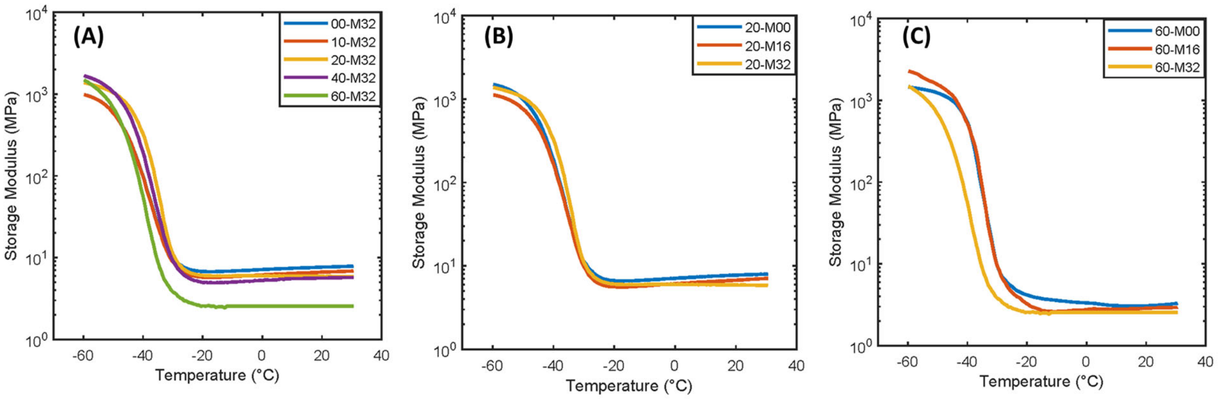

Figure S3. Storage modulus measured at rubbery plateau for series of PEGDA membranes varying (**A**) pre-polymerization water content at 32 mol% PEGMA (**B**) PEGMA mol% at 20 wt.% pre-polymerization water content, and (**C**) PEGMA mol% at 60 wt.% pre-polymerization water content.

Table S4. Glass transition temperature (T_g) of all the PEGDA-PEGMA membranes.

Membrane	T_g (°C) [DSC]	T_g (°C) [DMA]
00–M32	−42	−33
10–M32	−40	−33
20–M32	−42	−32
40–M32	−42	−32
60–M32	−43	−35
20–M00	−39	−31
20–M16	−41	−32
60–M00	−43	−32
60–M16	−39	−34

Table S5. Diffusive permeabilities of PEGDA based films varying pre − polymerization water wt% and PEGMA mol% to MeOH and NaOAc in single and two-solute measurements.

Membrane	PEGMA (mol%)	Single Solute in Feed Cell		Both Solutes in Feed Cell	
		MeOH ($\times 10^7$ cm^2/s)	NaOAc ($\times 10^7$ cm^2/s)	MeOH ($\times 10^7$ cm^2/s)	NaOAc ($\times 10^7$ cm^2/s)
00−M32	32	08.13 ± 0.10	0.75 ± 0.01	7.92 ± 0.25	0.82 ± 0.001
10−M32	32	10.20 ± 0.14	0.96 ± 0.17	9.84 ± 0.29	0.87 ± 0.002
20−M32	32	11.80 ± 0.42	1.72 ± 0.08	10.30 ± 0.09	1.49 ± 0.030
40−M32	32	17.10 ± 0.50	3.52 ± 0.07	16.50 ± 0.80	3.30 ± 0.020
60−M32	32	31.70 ± 1.14	10.1 ± 0.50	28.70 ± 0.43	8.15 ± 0.400
20−M00	00	10.90 ± 0.13	1.24 ± 0.40	9.46 ± 0.06	1.30 ± 0.020
20−M16	16	11.00 ± 0.50	1.32 ± 0.20	8.60 ± 0.34	1.43 ± 0.300
60−M00	00	29.00 ± 3.00	4.95 ± 0.31	26.70 ± 2.00	7.50 ± 0.260
60−M16	16	30.70 ± 0.83	8.30 ± 0.30	28.70 ± 0.83	7.80 ± 0.200

Table S6. Diffusive permeabilities of PEGDA based films varying pre − polymerization water wt% and PEGMA mol% to MeOH and NaOFm in single and two-solute measurements.

Membrane	PEGMA (mol%)	Single Solute in Feed Cell		Both Solutes in Feed Cell	
		MeOH ($\times 10^7$ cm^2/s)	NaOFm ($\times 10^7$ cm^2/s)	MeOH ($\times 10^7$ cm^2/s)	NaOFm ($\times 10^7$ cm^2/s)
00−M32	32	08.13 ± 0.10	0.89 ± 0.02	7.35 ± 0.06	1.13 ± 0.02
10−M32	32	10.20 ± 0.14	1.34 ± 0.09	9.76 ± 0.11	1.38 ± 0.09
20−M32	32	11.80 ± 0.42	2.56 ± 0.30	10.60 ± 0.20	2.69 ± 0.30
40−M32	32	17.10 ± 0.50	4.70 ± 0.30	17.30 ± 0.20	5.55 ± 0.26
60−M32	32	31.70 ± 1.14	10.9 ± 0.40	25.50 ± 0.30	12.70 ± 0.41
20−M00	00	10.90 ± 0.13	1.49 ± 0.20	7.56 ± 0.40	1.60 ± 0.08
20−M16	16	11.00 ± 0.50	2.05 ± 0.30	10.10 ± 0.13	1.97 ± 0.40
60−M00	00	29.00 ± 3.00	6.50 ± 1.00	28.40 ± 3.00	12.20 ± 0.22
60−M16	16	30.70 ± 0.83	10.3 ± 0.64	24.60 ± 0.30	11.40 ± 0.22

Table S7. Diffusive permeabilities of PEGDA based films varying pre − polymerization water wt% and PEGMA mol% to EtOH and NaOAc in single and two-solute measurements.

Membrane	PEGMA (mol%)	Single Solute in Feed Cell		Both Solutes in Feed Cell	
		EOH ($\times 10^7$ cm^2/s)	NaOAc ($\times 10^7$ cm^2/s)	EOH ($\times 10^7$ cm^2/s)	NaOAc ($\times 10^7$ cm^2/s)
00−M32	32	4.93 ± 0.01	0.75 ± 0.01	4.87 ± 0.02	0.69 ± 0.01
10−M32	32	6.22 ± 0.10	0.96 ± 0.17	6.02 ± 0.33	0.95 ± 0.02
20−M32	32	8.69 ± 0.05	1.72 ± 0.08	8.57 ± 0.16	1.68 ± 0.01
40−M32	32	11.90 ± 0.40	3.52 ± 0.07	11.20 ± 0.38	3.52 ± 0.14
60−M32	32	19.40 ± 0.52	10.1 ± 0.50	16.10 ± 0.37	6.70 ± 0.12
20−M00	00	4.64 ± 0.08	1.24 ± 0.40	5.27 ± 0.09	1.55 ± 0.20
20−M16	16	4.87 ± 0.05	1.32 ± 0.20	5.13 ± 0.01	1.52 ± 0.01
60−M00	00	14.90 ± 0.30	4.95 ± 0.31	17.4 ± 0.74	9.87 ± 0.08
60−M16	16	18.10 ± 0.53	8.30 ± 0.30	18.00 ± 0.62	8.02 ± 0.40

Table S8. Diffusive permeabilities of PEGDA based films varying pre − polymerization water wt% and PEGMA mol% to EtOH and NaOFm in single and two-solute measurements.

Membrane	PEGMA (mol%)	Single Solute in Feed Cell		Both Solutes in Feed Cell	
		EOH ($\times 10^7$ cm^2/s)	NaOFm ($\times 10^7$ cm^2/s)	EOH ($\times 10^7$ cm^2/s)	NaOFm ($\times 10^7$ cm^2/s)
00−M32	32	4.93 ± 0.01	0.89 ± 0.02	4.92 ± 0.05	0.95 ± 0.07
10−M32	32	6.22 ± 0.10	1.34 ± 0.09	6.18 ± 0.03	1.32 ± 0.08
20−M32	32	8.69 ± 0.05	2.56 ± 0.30	8.96 ± 0.07	2.72 ± 0.07
40−M32	32	11.90 ± 0.40	4.70 ± 0.30	11.9 ± 0.57	4.83 ± 0.50
60−M32	32	19.40 ± 0.52	10.9 ± 0.40	19.7 ± 0.21	12.4 ± 0.40
20−M00	00	4.64 ± 0.08	1.49 ± 0.20	5.25 ± 0.20	1.75 ± 0.01
20−M16	16	4.87 ± 0.05	2.05 ± 0.30	5.04 ± 0.12	1.84 ± 0.05
60−M00	00	14.90 ± 0.30	6.50 ± 1.00	15.8 ± 0.20	9.44 ± 0.20
60−M16	16	18.10 ± 0.28	10.3 ± 0.64	17.1 ± 0.45	10.50 ± 0.30

Table S9. True and Ideal Selectivity for Methanol (MeOH) and Ethanol (EtOH) over Sodium Formate (NaOFm).

Membrane	MeOH			EtOH		
	NaOFM (Ideal)	NaOFM (True)	% Difference	NaOFM (Ideal)	NaOFM (True)	% Difference
00−M32	9.07	6.76	28	5.50	5.17	6
10−M32	7.61	7.07	7	4.64	4.41	5
20−M32	4.61	4.25	8	3.39	3.29	3
40−M32	3.64	3.11	14.3	2.53	2.46	3
60−M32	2.91	2.00	31	1.78	1.59	10.7

Table S10. True and Ideal Selectivity for Methanol (MeOH) and Ethanol (EtOH) over Sodium Acetate (NaOAc).

Membrane	MeOH			EtOH		
	NaOAc (Ideal)	NaOAc (True)	% Difference	NaOAc (Ideal)	NaOAc (True)	% Difference
00−M32	10.80	9.71	10.5	6.57	7.09	8
10−M32	10.60	11.31	7	6.64	6.35	2
20−M32	6.86	6.91	3	5.05	5.10	1
40−M32	4.86	5.00	14.3	3.38	3.18	6
60−M32	3.14	3.52	12.2	1.92	2.40	25.1

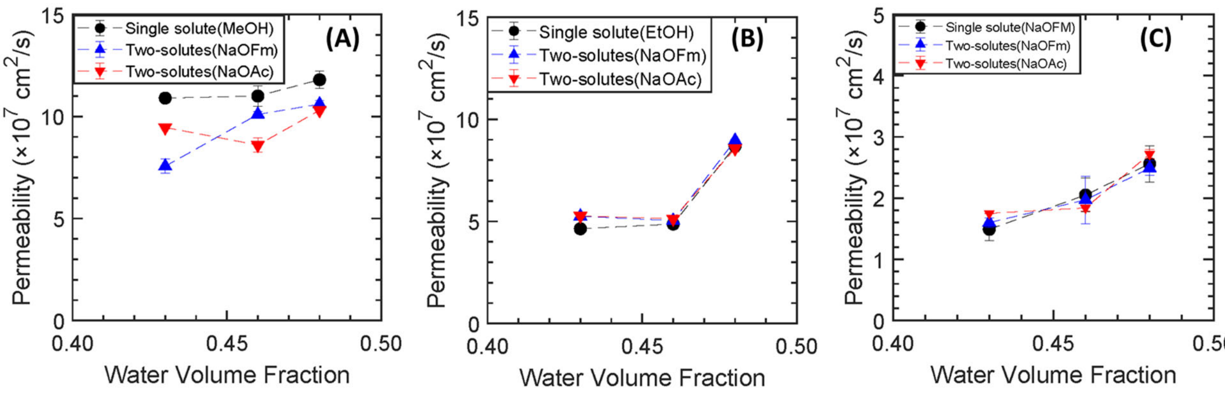

Figure S4. PEGDA-PEGMA (0, 16, 32 mol%) permeability to (**A**) methanol (MeOH), (**B**) ethanol (EtOH) (**C**) sodium formate (NaOFm), in single and two-solute vs. varied PEGMA mol% at 20% pre-polymerization water wt%.

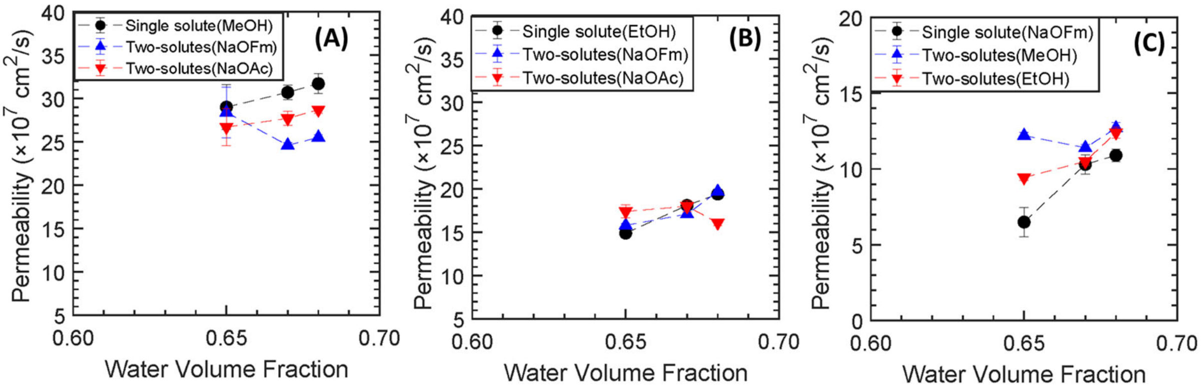

Figure S5. PEGDA-PEGMA (0, 16, 32 mol%) permeability to (**A**) methanol (MeOH), (**B**) ethanol (EtOH) (**C**) sodium formate (NaOFm), in single and two-solute vs. varied PEGMA mol% at 60% pre-polymerization water wt%.

References

1. Kim, J.M.; Dobyns, B.M.; Zhao, R.; Beckingham, B.S. Multicomponent transport of methanol and acetate in a series of crosslinked PEGDA-AMPS cation exchange membranes. *J. Membr. Sci.* **2020**, *614*, 118486, https://doi.org/10.1016/j.memsci.2020.118486.
2. Kim, J.M.; Beckingham, B.S. Comonomer effects on co-permeation of methanol and acetate in cation exchange membranes. *Eur. Polym. J.* **2021**, *147*, 110307, https://doi.org/10.1016/j.eurpolymj.2021.110307.
3. Kim, J.M.; Mazumder, A.; Li, J.; Jiang, Z.; Beckingham, B.S. Impact of PEGMA on transport and co-transport of methanol and acetate in PEGDA-AMPS cation exchange membranes. *J. Membr. Sci.* **2022**, *642*, https://doi.org/10.1016/j.memsci.2021.119950.
4. Kamcev, J.; Paul, D.R.; Freeman, B.D. Equilibrium ion partitioning between aqueous salt solutions and inhomogeneous ion exchange membranes. *Desalination* **2018**, *446*, 31–41, https://doi.org/10.1016/j.desal.2018.08.018.
5. Kamcev, J.; Paul, D.R.; Freeman, B.D. Ion Activity Coefficients in Ion Exchange Polymers: Applicability of Manning's Counter-ion Condensation Theory. *Macromolecules* **2015**, *48*, 8011–8024, doi:10.1021/acs.macromol.5b01654.
6. Kamcev, J.; Paul, D.R.; Manning, G.S.; Freeman, B.D. Ion Diffusion Coefficients in Ion Exchange Membranes: Significance of Counterion Condensation. *Macromolecules* **2018**, *51*, 5519–5529, doi:10.1021/acs.macromol.8b00645.
